# A non-destructive coconut fruit and seed traits extraction method based on Micro-CT and deeplabV3+ model

**DOI:** 10.3389/fpls.2022.1069849

**Published:** 2022-12-06

**Authors:** Lejun Yu, Lingbo Liu, Wanneng Yang, Dan Wu, Jinhu Wang, Qiang He, ZhouShuai Chen, Qian Liu

**Affiliations:** ^1^ School of Biomedical Engineering, Hainan University, Haikou, China; ^2^ Wuhan National Laboratory for Optoelectronics, Britton Chance Center for Biomedical Photonics, Key Laboratory of Ministry of Education for Biomedical Photonics, Department of Biomedical Engineering, Huazhong University of Science and Technology, Wuhan, China; ^3^ National Key Laboratory of Crop Genetic Improvement, National Center of Plant Gene Research, Huazhong Agricultural University, Wuhan, China

**Keywords:** plant phenomics, Micro-CT, coconut phenotypic traits, deep learning, non-destructive

## Abstract

With the completion of the coconut gene map and the gradual improvement of related molecular biology tools, molecular marker-assisted breeding of coconut has become the next focus of coconut breeding, and accurate coconut phenotypic traits measurement will provide technical support for screening and identifying the correspondence between genotype and phenotype. A Micro-CT system was developed to measure coconut fruits and seeds automatically and nondestructively to acquire the 3D model and phenotyping traits. A deeplabv3+ model with an Xception backbone was used to segment the sectional image of coconut fruits and seeds automatically. Compared with the structural-light system measurement, the mean absolute percentage error of the fruit volume and surface area measurements by the Micro-CT system was 1.87% and 2.24%, respectively, and the squares of the correlation coefficients were 0.977 and 0.964, respectively. In addition, compared with the manual measurements, the mean absolute percentage error of the automatic copra weight and total biomass measurements was 8.85% and 25.19%, respectively, and the adjusted squares of the correlation coefficients were 0.922 and 0.721, respectively. The Micro-CT system can nondestructively obtain up to 21 agronomic traits and 57 digital traits precisely.

## Introduction

Coconut (coconut, Cocos nucifera L.) is a perennial oil and food energy crop grown in humid tropical regions widely distributed in coastal areas within 20° north-south latitude. It is the most widely grown and used palm family in the world (Perera et al., 2009). In recent decades, research on coconut has developed rapidly (Lu and Liu (2021). The coconut transcriptome sequencing was completed by the Coconut Research Institute of the Chinese Academy of Tropical Agricultural Sciences in 2013 (Fan et al., 2013). Xiao Yong et al. completed the genome map of coconut in 2017, with a total of 28,889 protein-coding genes found (Xiao et al., 2017). Since then, molecular biology research related to coconuts, such as the development of SSR markers (Wu et al., 2019b) and the cloning and function characterization of specific genes, has been increasing (Yuan et al., 2015), providing powerful auxiliary methods for coconut breeding. With the completion of coconut genome sequencing and the gradual improvement of related molecular biology tools, molecular marker-assisted selection (MAS) for coconut has become possible. With the rapid development of functional genomics and molecular breeding, the ability to quickly screen a vast number of samples for targeted phenotypic traits is important (Fiorani et al., 2013). Manual phenotyping methods of coconut traits, such as weighting, dehusking, and measuring the central axis, minor axis, and the perimeter, are time-consuming, labor-intensive, and potentially damaging, and so hinder the development of coconut genomics (Furbank and Tester, 2011).

Over the past few decades, many phenotyping systems have been developed for coconut trait extraction and analysis. Koffi et al. compared the differences in morphological and agronomic traits of two coconut samples obtained from *in vitro* culture and seed germination. They verified that the plant height and root length of the plants cultured *in vitro* were significantly smaller in the first one to two years, but there was no significant difference after more than five years (Koffi et al., 2013). Carvalho et al. described the spatial distribution of natural infection of coconut resin deposition disease in commercially available orchards and its progression over time (Carvalho et al., 2013). Thomas et al. proposed an automatic coconut quality rating method based on sound processing technology, which mainly judges whether the coconut has sufficient water content or is damaged and rotted (Thomas and Anita, 2017). Caladcad et al. used an artificial neural network (ANN), random forest (RF), and support vector machine (SVM) to classify coconut maturity according to sound signals. The coconuts are divided into three categories: immature, mature, and over mature. Among the three methods, the random forest method obtained the highest classification accuracy of 83.48% (Caladcad et al., 2020). Beevi et al. used mites to infect coconut seeds and found that the mites did not reduce the germination rate of coconut seeds, but the mites invaded coconut. When the seed coat area is more than 25%, the leaf area of coconut seedlings will be significantly reduced (Beevi et al., 2006). Yang et al. Used iTRAQ to conduct proteomic analysis of coconuts under cold stress to understand the molecular mechanisms of tropical crops adapting to cold stress and analysis the change of specific proteins in cold-sensitive and cold-resistance varieties (Yang et al., 2020). Anant et al. Studied coconut endosperm with a low-temperature scanning electron microscope. The distribution and structure of proteins, phospholipids, and glycolipids at the oil-liquid interface were described, and the mechanism of oil bodies maintaining stability in the endosperm was revealed (Dave et al., 2019).

These studies use optical techniques such as visible light imaging, microscopic imaging and low-temperature electron microscopy, combined with chemical analysis, artificial evaluation, acoustic analysis and other methods to obtain phenotyping traits. The coconut surface structure, color, and spectral information have been successfully obtained. However, there are certain deficiencies in the acquisition of the internal structure information of the coconut, which usually requires the destruction of the coconut shell (Duliu, 1999). Moreover, most of the work can only obtain 2D image data, but many agronomy traits require analysis in a 3D model.

With the continuous development of technology and the reduction of costs, CT technology has gradually expanded from specialized medical examination methods to other biological detection fields. It is increasingly used by plant research laboratories for the detection of macro, micro, and even nanoscale levels of phenotypes in plants (Piovesan et al., 2021). Root measurement systems like the RootViz3D^®^ (Tracy et al., 2012) and the VGStudioMAX^®^ (Tracy et al., 2015) provide non-destructive, high-resolution 3D reconstruction methods to reveal the internal structure of plant tissues and organs. Moreover, The Micro-CT system is also widely used in the morphological detection of stems and lignin and vascular bundles in stems (Wu et al., 2019a) (Brodersen et al., 2011) (Wu et al., 2019a), and the extraction of flowers (Dhondt et al., 2010), leaf (Stuppy et al., 2003) and grain traits (Jhala and Thaker, 2015).

In summary, the current research on coconut phenotype detection mainly involves two aspects, acquiring coconut surface information by destructive detection which provides a fast and convenient phenotyping approach, or exploring the detailed microstructure using relatively high-cost equipment. Both ways provide plant trait data for researchers. The CT reconstruction technology, image processing, and deep learning have been widely used in the extraction of other crops, and related technologies can be transferred to the extraction of coconut traits. In the present work, we developed a non-destructive traits measurement system based on a Micro-CT that can obtain the 3D model of coconut fruits. The system can measure up to 21 agronomic traits and 57 digital traits with a high spatial resolution (up to 50 μm), which provides a non-destructive coconut in-shell trait analysis and 3D model reconstruction for breeders. A total of 120 coconut fruits and 40 coconut seeds were phenotyped non-destructively. In addition, a DeepLabV3+ model was trained for slice image segmentation.

## Method

### Material and experimental design

In this study, 120 coconut fruits and 40 coconut seeds were subjected to micro-CT, structured light 3D reconstruction, and manual measurements. Among the 120 coconuts fruits, 60 were “Wanning golden” coconuts, and 60 were “Wenye 4” coconuts. The 40 coconut seed samples were all dehusked “Wenye 4” coconuts at different germination stages.

The coconut fruit samples were firstly weighted to obtain the total weight and then measured using a structured-light 3D scanner to obtain the 3D model of the fruit surface. A Micro-CT system processed all the samples to capture the X-ray projection image. After imaging, the mesocarp was removed to get the dehusked coconut and was weighted to get the dehusked fresh biomass. The dehusked coconut seeds samples were processed using a Micro-CT system without structured-light 3D scanner measurement since the surface texture was too complicated for structured-light scanning. At last, the shell was broke open to measure the mass of the coconut liquid and solid albumen.

### Main components and configuration of Micro-CT

**Figure 1:**
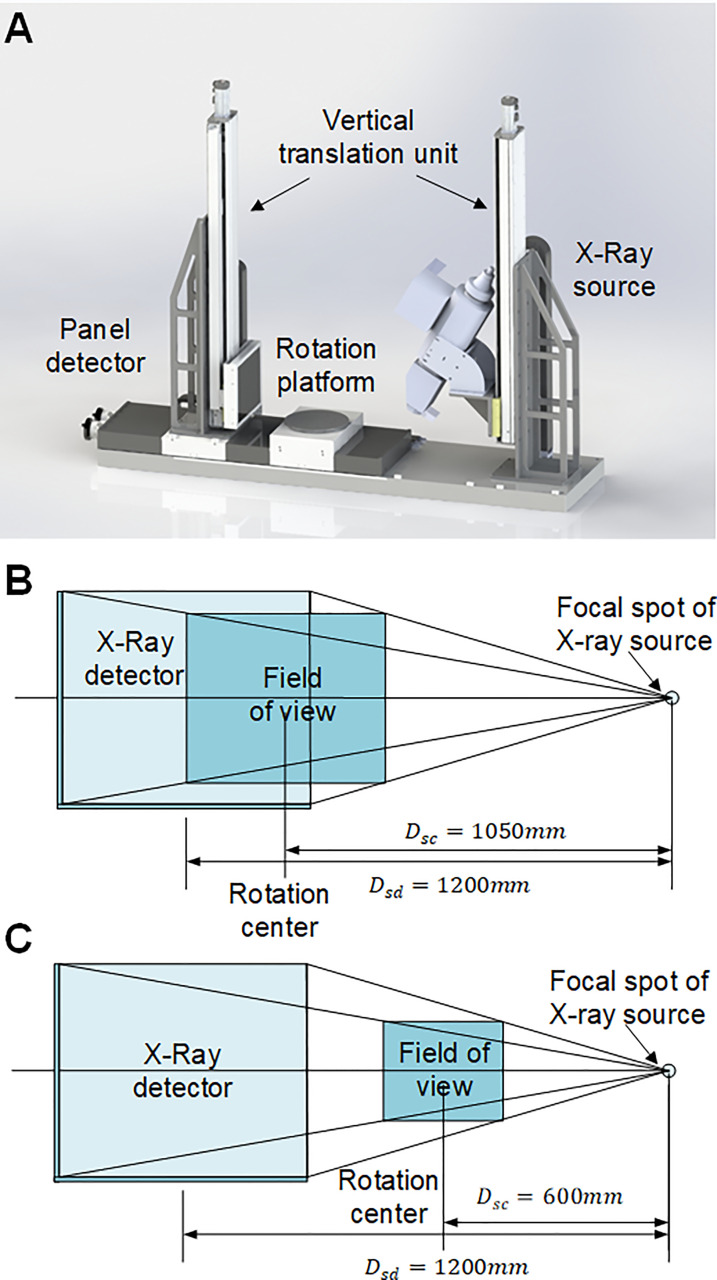
Principal components and configuration of Micro-CT system. **(A)** Hardware design of the Micro-CT system. **(B)** The field of view for coconut fruit imaging. The active area of the panel detector is 248mm⨯298mm with a resolution of 2512⨯3008 pixels. The distance between the X-ray source and the detector (DSD) is 1200 mm, the distance between the X-ray source and the rotation center (Dsc) is 1050 mm; thus, the field of view is 217mm⨯260mm, and the spatial resolution can be calculated as 86μm⨯86μm. **(C)** The field of view for coconut seed imaging. The distance between the X-ray source and the rotation center (DSC) is 600 mm; thus, the field of view is 124mm⨯149mm, and the spatial resolution can be calculated as 49μm⨯49μm.

The Micro-CT imaging system was developed to obtain CT projection images non-destructively. The Micro-CT consists of six main elements: an X-ray source (X-RAY WorX GmbH, German), an X-ray source chiller (Nova600, OXFORD, UK), an X-ray flat panel detector (XRD 3025, Varex, USA), a rotation platform (MSMD022G1U, Panasonic, Japan), a lead chamber, a computer (CPU i5-7700k), and a PLC controller (CP1H, OMRON Corporation, Japan). **Figure 1A** shows the hardware design and layout of the Micro-CT system. The spacial configuration of the Micro-CT system is provided in **Figures 1B, C**. For coconut fruit measurement, since the projection size of coconut fruit is close to the maximum imaging active area, the rotation platform was set against the X-ray panel detector and obtained a spatial projection resolution of 86μm and a FOV of 217mm⨯260mm at the rotation axis. For coconut seed measurement, the trade-off between the CT image resolution and CT scan area was set to ensure a whole seed scan with a spatial projection resolution of 49μm and a FOV of 124mm ×149mm at the rotation axis.

### Image acquisition and pre-reconstruction process of Micro-CT

The control program of the system was developed based on the XISL (X-ray Imaging Software Library) provided by PerkinElmer for panel detector control, the X-COM API provided by X-RAY WorX GmbH for X-Ray source control, and the OpenCV library for image process.

The image-acquisition program continuously captures the X-Ray projection image with a refresh rate of 180ms per frame. A black-white current calibration was applied when the frame was transmitted. 360 projection images were captured for each sample with a step of 1° and a total angle of 360°. The rotation platform will stop for 2.7 seconds for each step to capture 15 frames, and the 15 frames within the same step were averaged to reduce the influence of noise in cone-beam CT. A difference detection algorithm was also developed to detect the keyframe before and after the rotation occurred.

### Overview of the Micro-CT image process procedure

In this study, we developed an application for CT projected image acquisition, CT section image reconstruction, fruit 3D model reconstruction, and fruit traits extraction combined. The critical steps of the image process include projection image calibration, section image reconstruction, section image segmentation, fruit 3D model reconstruction, and fruit phenotypic traits calculation.

Considering the limitations of the circuit design and manufacturing process of the panel detection, a calibration of the projection images is essential. The actual signal of the projection image is calculated with


$$G=\ln(\frac{S_{0}-S_{B}}{S_{W}-S_{B}})$$

Where G is the resulting signal, *S*_0_, *S*_*B*_  and *S*_*W*_ is the original signal, the black current, and the white current separately.

**Figure 2:**
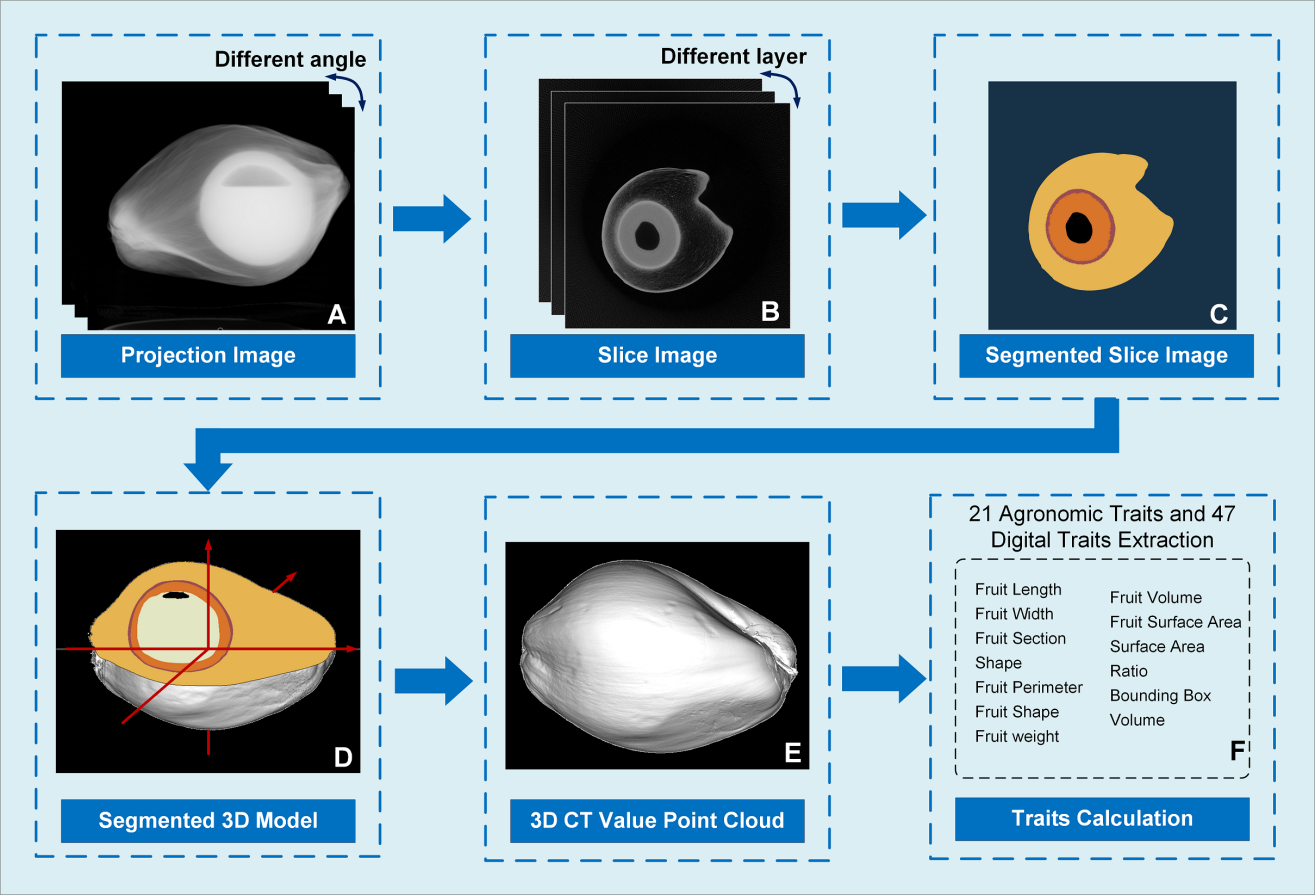
Image analysis pipeline of Micro-CT system. **(A)** As the coconut sample rotated, 360 X-ray projected images at different angles were acquired and calibrated; **(B)** A FDK algorithm was applied to obtain a reconstructed section image of the coconut fruit; **(C)** A DeepLabV3+ model was used to segment the slice image and acquire the area of the mesocarp, endocarp, solid and liquid albumen and cavity; **(D)** A local least squares and a Poisson surface reconstruction algorithm were introduced to convert the boundary points into a 3D model; **(E)** Filled the 3D model to obtain the 3D cloud point with CT value; **(F)** Using the segmentation results of the slice images, the 3D model was segmented.

The projection image calibration results in multiple angles are shown in **Figure 2A**. An FDK algorithm (Feldkamp et al., 1984) was applied to reconstruct the section images in multiple layers of the coconut fruit and seed from 360 calibrated projection images (**Figure 2B**). The slice images were segmented using A DeepLabV3+ model with an Xception backbone to determine the area of mesocarp, endocarp, solid albumen, liquid albumen, and cavities in different layers (**Figure 2C**). Coordinates of the boundary pixels between each segmented part in the slice images and the height coordinates of each layer were combined to obtain the boundary 3D point cloud. Triangulation of the surface points was performed using the local least-squares algorithm. After calculating the normal vector direction of the boundary points, the Poisson surface reconstruction algorithm (Kazhdan et al., 2006) is applied to obtain the segmented three-dimensional network model (**Figure 2D**). The 3D model was then filled with the CT value of each voxel to obtain the 3D CT value point cloud (**Figure 2E**). The CT value are specified with the air set to 0 and the water set to 200. Then all the pixels in the slice images can be conveniently transformed into a grayscale image(lower than 255) for later segmentation. According to the segmented 3D model and the 3D cloud point with CT value, phenotypic traits of the epicarp, mesocarp, solid albumen, liquid albumen, and cavity were calculated (**Figure 2F**).

### Use DeeplabV3+ with the Xception backbone for segmentation

**Figure 3:**
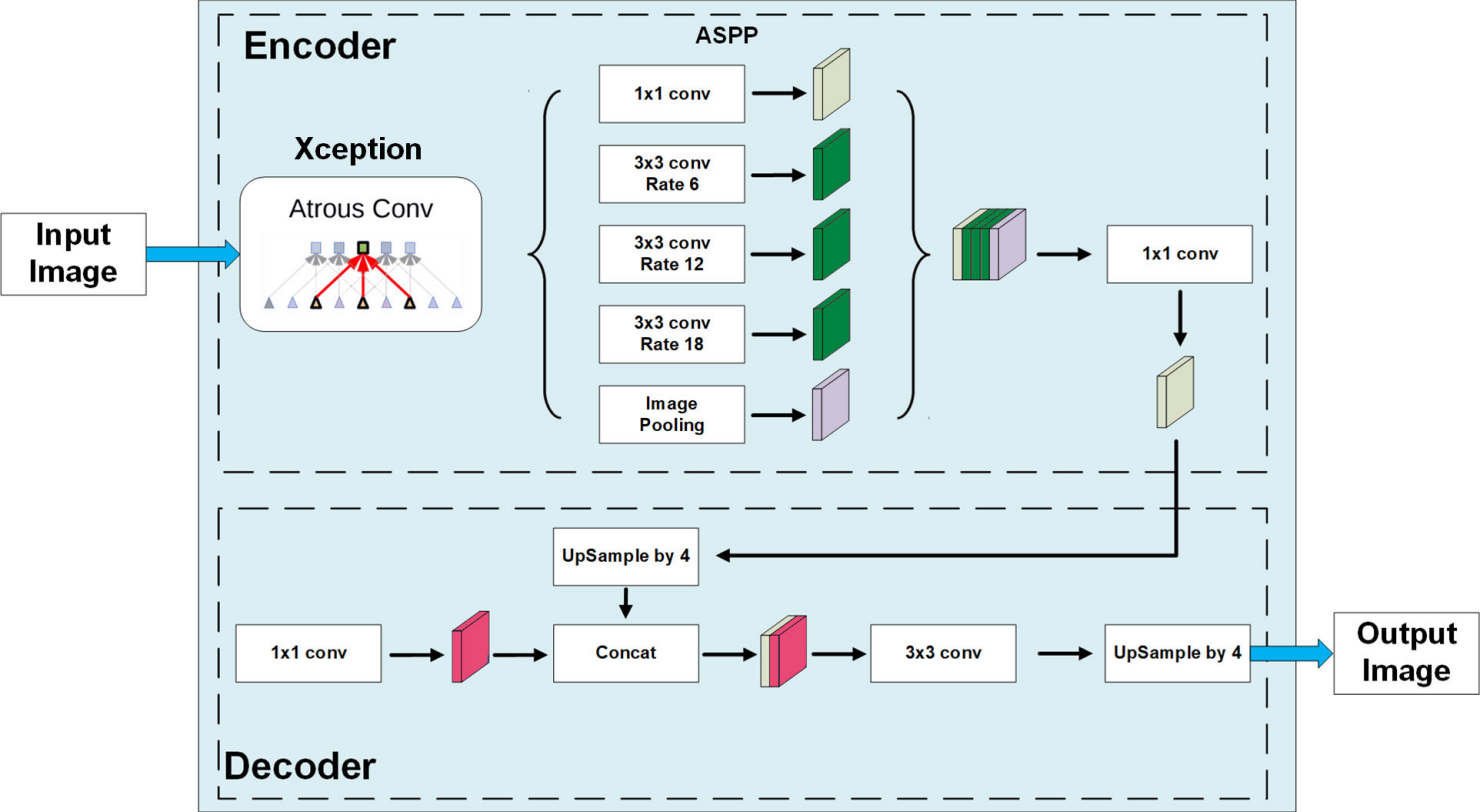
The model structure of DeepLabV3+ with the Xception backbone.

Section image segmentation is one of the critical steps in the image process of the Micro-CT system. We introduced the DeepLabV3+ model with an Xception backbone to obtain segmented results of the image. DeepLabV3+ is a convolutional neural network model designed for pixel-based semantic image segmentation (Chen et al., 2018). Several kinds of backbones can be used in DeepLab, including ResNet (He et al., 2015), Xception (Chollet, 2017), and MobileNet (Howard et al., 2017). The Xception model was selected for its high accuracy. The implementation, training, and evaluation were processed using TensorFlow (Martín Abadi et al., 2015). The transfer training was started with an initialized model pretrained on the VOC 2012 dataset. The loss weight of loss function is modified according to the pixel numbers of each part. The logit and output layers are excluded to train the model on our dataset. **Figure 3** displays the model structure of DeepLabV3+.

The training data set includes slice images from 100 coconuts selected from the samples. A balanced sample including coconuts with no solid albumen, thin solid albumen, thick solid albumen, and with or without cavities was selected to improve the representativeness of the selected samples. There are a total of 217,660 tomograms reconstructed from these coconut samples. Considering that the differences between adjacent layers are slight, slice images were taken every 20 layers for sampling. that is, 10,883 slice images are used as original training samples, and the actual spatial distance between each adjacent image is 2mm. Considering that the outline of the transverse section of the coconut is approximately circular without directionality, the horizontal-fliped, vertical-fliped, clockwise-rotated, and counterclockwise-rotated images were generated and included in the training data set for data augmentation. The final data includes 54,415 tomograms and corresponding annotation images.

**Figure 4:**
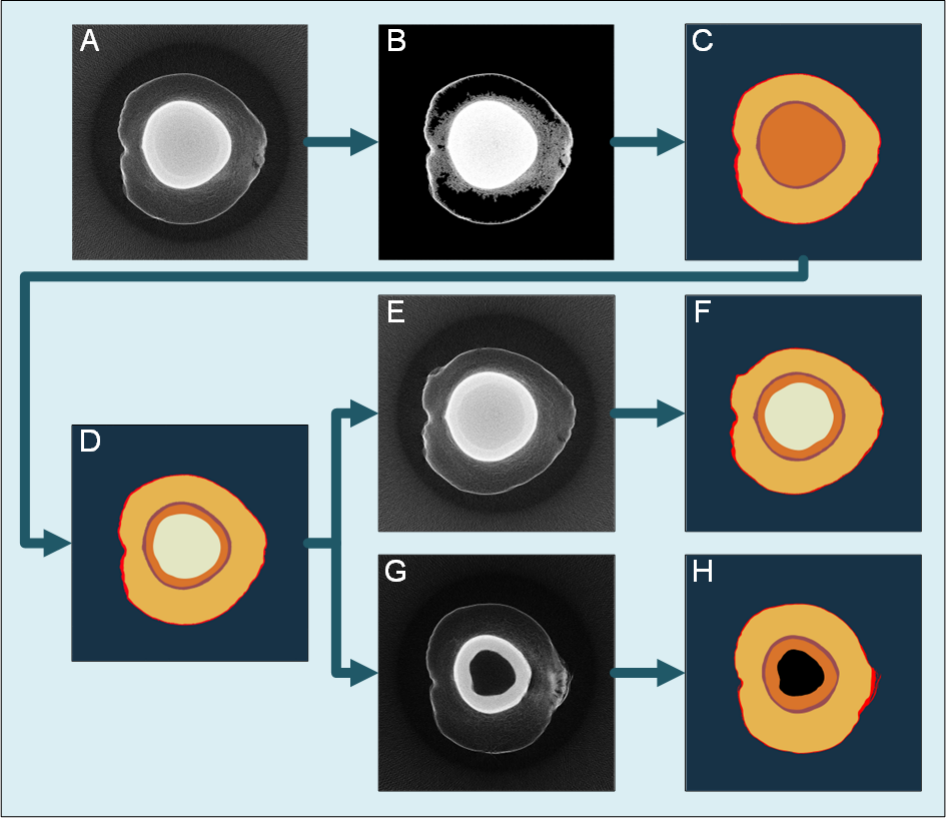
flow chart of traditional image processing segmentation algorithm. **(A)** The slice image at the middle height of the sample. **(B)** The background area is removed by the Otsu algorithm and connected component algorithm. **(C)** The edge enhancement algorithm determines the coconut coat and inner shell area. **(D)** The seed points and threshold tolerance of solid and liquid albumen are manually set to obtain the segmentation result. **(E)** The slice image at another height close to **(A)**. **(F)** Take the segmentation result **(D)** as the template to segment **(E)**. **(G)** The slice image at another height far from **(A)**. **(H)** Take the segmentation result **(D)** as the template to segment **(G)**. *A priori* knowledge detects the cavity area at the center.

All tomographic images were segmented using a semi-automatic segmentation program based on a self-adapting threshold algorithm, region growing algorithm, and edge enhancement algorithm to reduce the labor cost of the manual labeling of the training set. The basic idea of the semi-automatic segmentation algorithm is to segment a slice image at the middle height with manually input seed points and thresholds and use this segmentation result as a template to segment the adjacent slices, which is shown in **Figure 4**. The slice image at the middle height usually contains various parts, including the solid and liquid albumen and the coconut shell (**Figure 4A**). An Otsu threshold algorithm extracts the exocarp and inner shell areas with a relatively high absorption rate, and a connected component is used to remove noise points (**Figure 4B**). Then, an edge enhancement algorithm based on linear fitting in polar coordinate is applied to determine the areas of the pericarp and the shell (**Figure 4C**). The boundary between solid and liquid albumen is blurred, and it is necessary to manually provide the seed point of solid and liquid albumen and the threshold tolerance (**Figure 4D**). The segmentation result is used as a template to segment adjacent layers using a region-growing algorithm (**Figures 4F, H**). Before training the model, the results were manually inspected and modified to ensure the samples had no apparent errors. The semi-automated labeling algorithm can reduce the labor consumption in creating the training set. Therefore, compared with other studies, this study has a larger training sample set. This semi-automated labeling algorithm can also be extended to other deep learning tasks whose sample pictures also contain large areas with similar gray scale to reduce the cost of the other scientific research.

### Traits measurements of the coconut

With the segmented section images, all the voxels of the epicarp, mesocarp, solid albumen, liquid albumen, and cavity can be located to establish the 3D model of the whole fruit. Up to 21 agronomic traits, including the fruit volume, surface area, major and minor axis, coconut solid and liquid albumen volume that can be directly obtained from the 3D model, and the total fruit weight, copra weight, and other traits through modeling, were measured together with 47 digital traits were measured. Volume-related traits, including the total fruit volume, total dehusked volume, solid albumen volume, liquid albumen volume, and shell volume, can be directly calculated from the 3D model. To estimate biomass traits, like the mesocarp biomass, the solid albumen biomass, and the liquid albumen biomass, several models, including linear, power, exponential, logarithmic, quadratic, and logistic, using phenotypic traits were built and compared. The definitions and abbreviations of the agronomic traits are shown in **Table 1** and the digital traits are shown in **Table 2**.

**Table 1:** The argronimic trait classification and abbreviation.

Trait classification	Trait	Trait abbreviation
Fruit traits	Fruit shape	FS
	Fruit longitudinal slice shape	FLS
	Fruit length	FL
	Endocarp thicknessFruit longitudinal perimeter	ETFLP
	Fruit width	FW
	Fruit perimeter	FP
Seed traits	Seed shape	SP
	Seed longitudinal slice shape	SLS
	Seed length	SL
	Seed longitudinal perimeter	SLP
	Seed Width Solid albumen thickness	SW SAT
Weight traits	Fruit total weight	FTW
	Seed total weight	STW
	Mesocarp weight	MW
	Copra weight	CW
	Liquid albumen weight	LAW
	Solid alnumen weight	SAW
	Solid albumen dry biomass	FDB
	Solid albumen dry biomass ratio	FDBR

**Table 2:** The digital trait classification and abbreviations.

Traits	Trait Abbreviation
Volume	Vol
Surface Area	Sa
Volumn Surface Area Ratio	VolSaR
Circumscribed Sphere Volumn	CSVol
Circumscibed Sphere Volumn Ratio	CSVolR
Inscribed Sphere Volumn	ISVol
Inscribed Sphere Volumn Ratio	ISVolR
Bounding Box Volumn	BBVol
Bounding Box Volumn Ratio	BBVolR
Bounding Box Length	BBL
Bounding Box Width	BBW
Bounding Box Height	BBH
Maximun Logitudinal Section Area	MLSA
Logitudinal Section Perimeter Area Ratio	LSPAR
Logitudinal Section Bounding Rectangle Height	LSBRH
Logitudinal Section Bounding Rectangle Width	LSBRW
Logitudinal Section Aspect Ratio	LSAR
Maximun Cross Section Area	MCSA
Cross Section Perimeter Area Ratio	CSPAR
Cross Section Bounding Rectangle Height	CSBRH
Cross Section Bounding Rectangle Width	CSBRW
Cross Section Bounding Rectangle Aspect Ratio	CSBRAR
Shell CT Intergral Value	SCTIV
Milk CT Intergral Value	MCITV
Shell Volumn	SVol
Shell Surface Area	SSa
Shell Volumn Surface Area Ratio	SVSAR
Shell Circumscribed Sphere Volumn	SCSVol
Shell Circumscibed Sphere Volumn Ratio	SCSVolR
Shell Inscribed Sphere Volumn	SISVol
Shell Inscribed Sphere Volumn Ratio	SISVolR
Shell Bounding Box Volumn	SBBV
Shell Bounding Box Volumn Ratio	SBBVR
Shell Bounding Box Length	SBBL
Shell Bounding Box Width	SBBW
Shell Bounding Box Height	SBBH
Shell Maximun Logitudinal Section Area	SMLSA
Shell Logitudinal Section Perimeter Area Ratio	SLSPAR
Shell Logitudinal Section Bounding Rectangle Height	SLSBRH
Shell Logitudinal Section Bounding Rectangle Width	SLSBRW
Shell Logitudinal Section Aspect Ratio	SLSAR
Shell Average Thickness	SAT
Copra Average Thickness	CAT
Copra Volumn	CVol
Milk Volumn	MVol
Coat CT Intergral Value	CCTIV
Copra CT Intergral Value	CCTIV

## Result

### Projection image obtained from Micro-CT system

**Figure 5:**
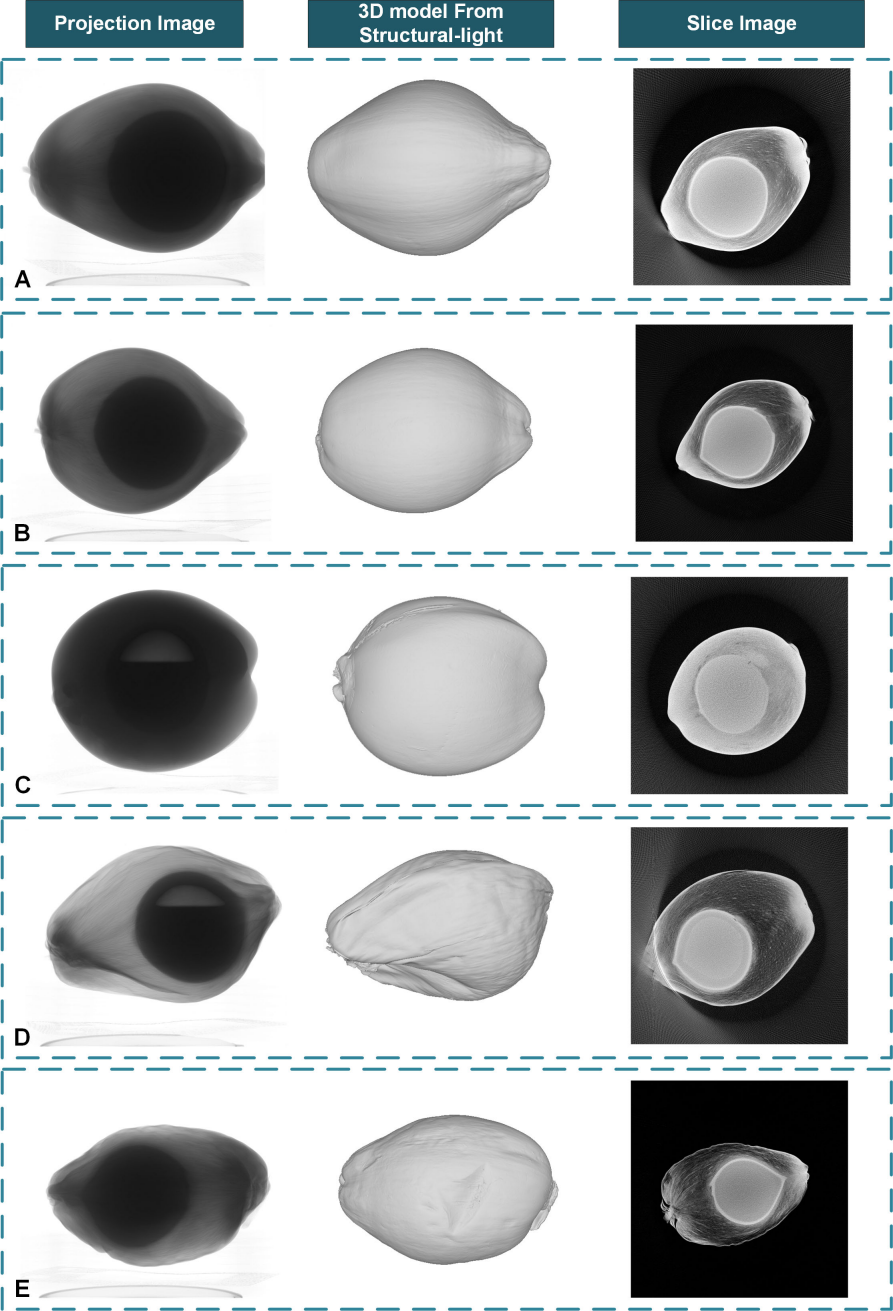
The coconut fruit projection images from the Micro-CT system, the corresponding 3D model from the structured light system, and the reconstructed slice images with some out-of-ranged samples. **(A)** An out-of-ranged immature sample with full liquid albumen. **(B)** A normal immature sample with full liquid albumen. **(C)** A normal immature sample with limited liquid albumen. **(D)** An out-of-ranged mature sample with limited liquid albumen. **(E)** A normal mature sample with full liquid albumen.

The pre-experimental results of CT reconstruction of a small number of samples show that with the tube voltage set to 120kV and the tube current set to 300µA, the projection images will have better contrast. To reduce the influence of the low signal-to-noise ratio of cone-beam CT, 15 frames were collected and averaged within the same angle step. The coconut fruit CT projection images and the corresponding 3D model acquired from the structured light system are shown in **Figure 5**. Samples A, B, and C are young coconuts with higher moisture content in mesocarp, and their X-Ray absorption rate is relatively high. Samples D and E are mature coconuts. These samples have lower water content and lower absorption rate of X-rays. In terms of fruit shape, samples A, D, and E are oblong-shaped, and samples B and C are oval-shaped. In terms of coconut milk content, samples A, B, and E were filled with coconut milk, while samples C and D had large cavities areas in the shell.

**Figure 6:**
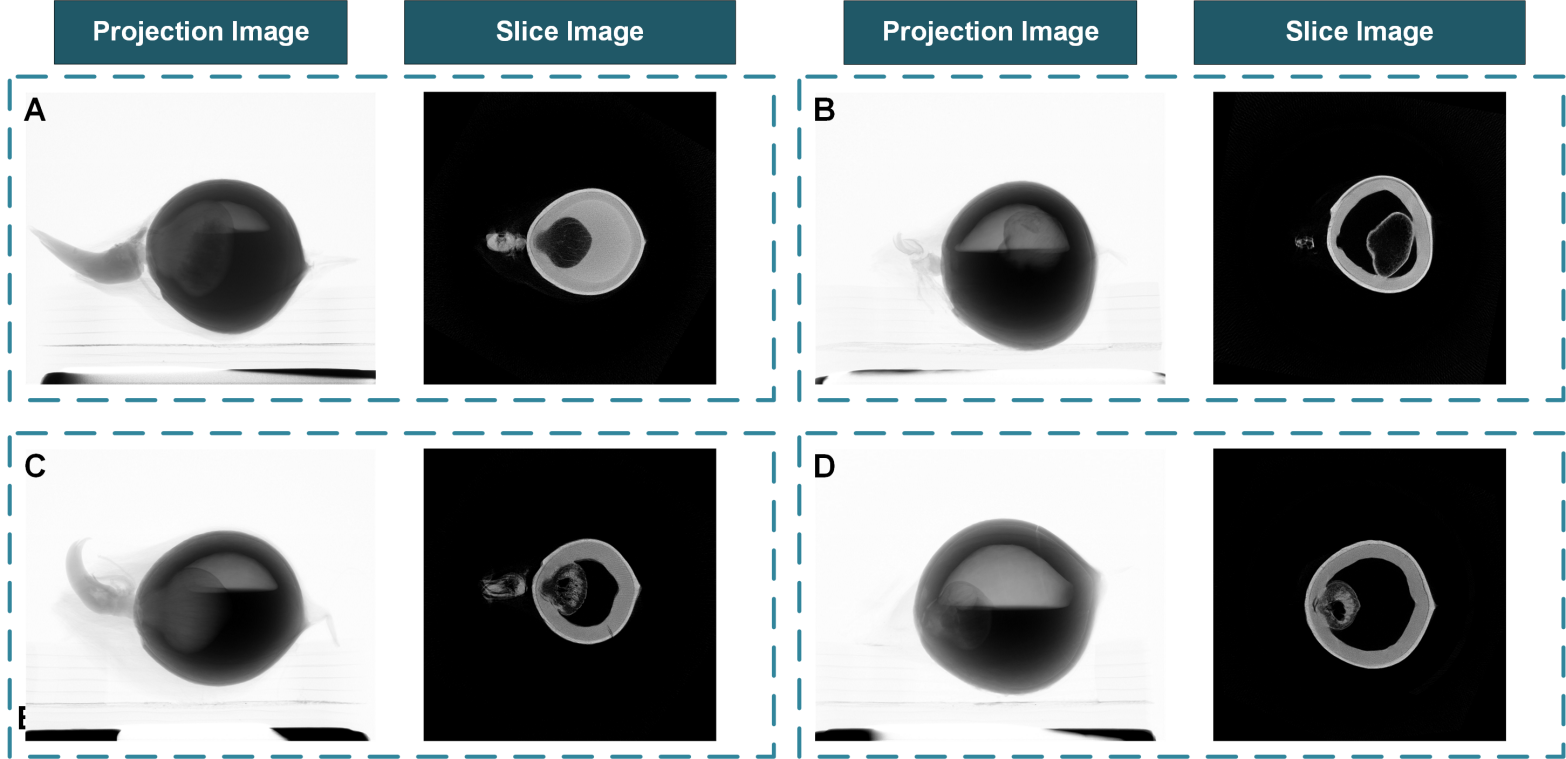
The coconut fruit projection images from the Micro-CT system and the reconstructed slice images. **(A)** A fast-growing sample with well-developed haustorium and sufficient albumen. **(B)** A sample that failed to germinate. **(C)** A normal sample with a developing haustorium and sufficient albumen. **(D)** A slow-growing sample with insufficient albumen.

The pre-experimental results show that with the tube voltage set to 80kV and the tube current set to 100µA, the projection images of coconut seeds will have better contrast. 15 frames were collected and averaged within the same angle step. The projection images and CT slice images of the coconut seeds are shown in **Figure 6**.

### Reconstructed slice image obtained from Micro-CT system

In this paper, we use a CUDA-based FDK algorithm to reconstruct the coconut fruit and seed slice images. For the coconut seed experiment, the actual size of the reconstruction area is set to 150mm⨯150mm for each layer with a resolution of 3000⨯3000 pixels, and about 3000 layers were reconstructed in the vertical direction with a slice image spatial resolution of 50 µm. For the coconut fruit experiment, the actual reconstruction area in the coconut fruit experiment is set to 300mm⨯300mm for each layer with a resolution of 3000⨯3000 pixels, and 3000 layers were reconstructed in the vertical direction with a spatial resolution of 100 µm per voxel. The results are displayed in **Figure 5**. The reconstruction results show that the CT system can obtain the distribution of tissues inside the coconut, such as epicarp, mesocarp, solid albumen, liquid albumen, and cavity can be observed from the tomogram. The mesocarp of young coconut with higher water content has a higher absorption rate and is more difficult for X-Ray to penetrate. The size of samples A and D exceeds the detector’s maximum imaging range. Thus the outermost edge of the sample cannot be observed in some of the projection images, resulting in an artifact at the corresponding position in the reconstructed slice images and affecting subsequent image processing. Samples A, B, and C were young coconuts with little solid or semisolid albumen, which is difficult to observe on the slice image. In comparison, mature samples D and E contain more solid albumen, which can be distinguished in the image.

### Slice image segmentation and the performance evaluation of DeepLabV3+ segmentation

The data was divided into training set, validation set and test set. In this paper, the training set and validation set are both derived from the 100 coconut samples in a ratio of 9:1, which contained 48974 and 5441 tomograms respectively. The test set was established from the remaining 20 coconut samples using the same slice image sample selection and data augmentation used in building the training set, and contained a total of 10027 tomograms.

**Figure 7:**
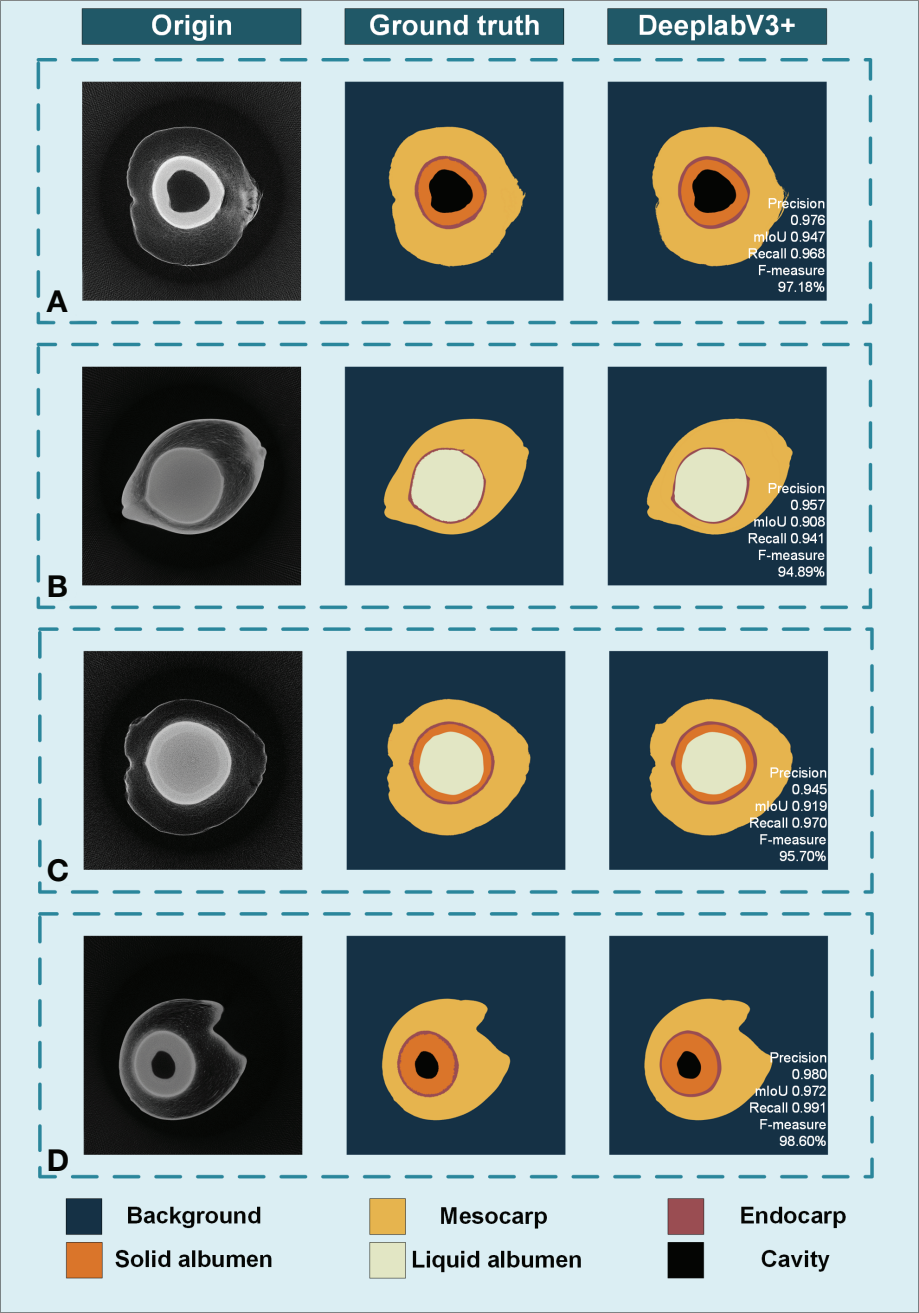
The accuracy analysis of DeepLabV3+ model. **(A)** Sample with cavities and solid albumen. **(B)** Sample without cavities and have thin solid albumen. **(C)** Sample with thick solid albumen. **(D)** Sample with thick solid albumen and cavities.

Four indicators, including average precision, average recall, F1 value, and average intersection, are used to evaluate the segmentation performance of the model. **Figure 7** shows the results of the DeepLabV3+ algorithm and the manual segmentation result. Samples A and D contain cavities and solid albumen. Sample B contains full liquid albumen with thin and gelatinous solid albumen that are difficult to distinguish from slice image, while sample C has relatively thick solid albumen.

For DeepLabV3+ model, the averages of Precision, Recall, F1-measure and IoU were 90.21%, 89.58%, 88.36% and 88.11%, respectively. In general, the DeepLabV3+ model has better segmentation results for tomograms containing different components in fruits of different stages and sizes.

### The acquisition of 3D segmented model and CT value point cloud

**Figure 8:**
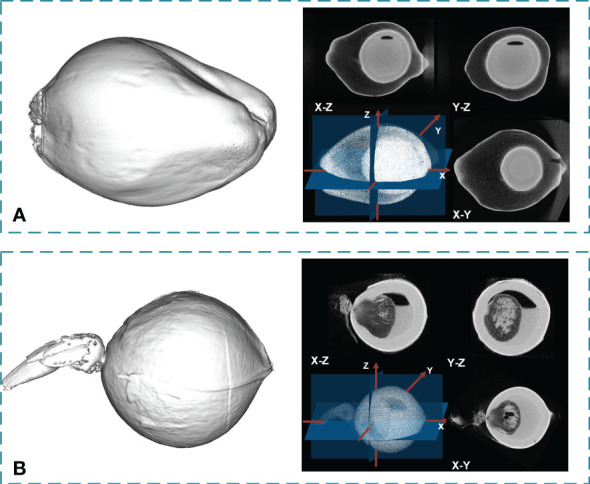
The 3D point cloud model of coconut fruit and seeds, and the sectional views in three directions. **(A)** Result for a coconut fruit sample. **(B)** Result for a coconut seed sample.

From the segmented slice images obtained from the DeepLabV3+ model, pixels of each part of the coconut can be located, and the coordinates of the boundary pixels between adjacent segmented parts were combined to establish the boundary 3D point cloud. Triangulation of the boundary points was performed using the local least-squares algorithm. After calculating the normal vector direction of the boundary points, the Poisson surface reconstruction algorithm is applied to obtain the segmented three-dimensional network model of the whole fruit. The 3D model was filled with the CT value of each voxel to obtain the 3D CT value point cloud to acquire a detailed segmented slice image of each part with CT value. Besides the transverse section images, the voxels in the cloud point were reorganized to achieve slice images in other directions. The 3D point cloud model of the coconut fruit is shown in **Figure 8A**, and the 3D point cloud model of the coconut seed is shown in **Figure 8B**.

### Accuracy evaluation of fruit volume and coconut milk measurement

Volume-related parameters, including fruit volume, coconut endocarp volume, coconut solid albumen volume, and coconut liquid albumen volume, can be directly measured from the segmented 3D model. The liquid albumen is usually called coconut milk in agricultural production. Milk yield can be directly obtained by measuring the volume in the 3D model. In the experiment, the fruit volume of 120 coconuts was measured using the Micro-CT system, and the results were compared with the model volume obtained by structured light measurement.

**Figure 9:**
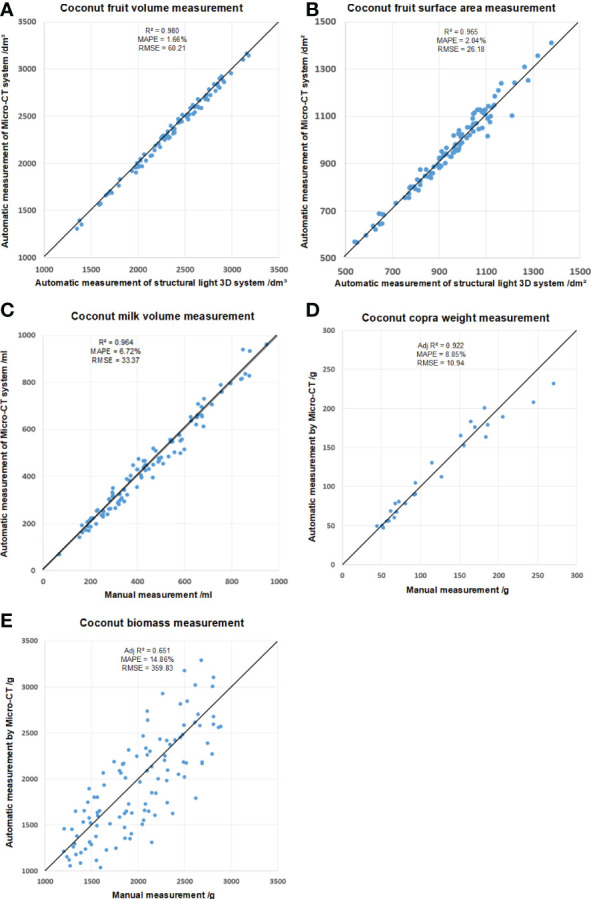
Mesurement of **(A)** coconut fruit total volume, **(B)** total surface area, **(C)** milk volume, **(D)** copra weight, and **(E)** biomass.

As shown in **Figures 9A, B**, the R^2^, MAPE and RMSE of the coconut fruit total volume measurement were 0.980, 1.66% and 60.21cm³, respectively. In addition, the R^2^, MAPE RMSE of coconut fruit surface were 0.965, 2.04% and 26.18cm², respectively.

The data obtained by the Micro-CT system has high accuracy in general. Compared with the data obtained by the structured light system, the volume calculated by Micro-CT is slightly smaller, and the surface area is slightly larger. It is speculated that the CT system is more sensitive to the pits on the coconut surface than the structured light 3D reconstruction system.

In addition, the volume of coconut liquid albumen was also measured in this experiment and compared with the value obtained by manual measurement. When measuring the volume of coconut liquid albumen manually, a funnel and a measuring cylinder were used for direct measurement, and three individual readings from three researchers were averaged. The result is shown in **Figure 9C**, and the R^2^, MAPE, and RMSE were 0.964, 6.72%, and 33.37mL, respectively.

### Accuracy evaluation of coconut copra measurement and biomass measurement

The coconut solid albumen is usually called coconut copra in agricultural production. The coconut copra content in the immature green coconut is minimal, with only a few grams of gelatinous copra in some samples, making it difficult to distinguish in the slice images. Therefore, in this paper, only samples with a coconut solid albumen content of more than 50g are considered, with totaling 26 samples.

Based on the tomogram obtained by the Micro-CT system and the segmentation results from the DeepLab V3+ model, the integral value of the CT value of the coconut voxel can be calculated. The copra weight were measured by modeling the CT value and some other morphological traits using SPSS software (Version 26.0, IBM Crop., Armonk, USA), as shown in **Figure 9D**. The Adjusted R^2^, MAPE, and RMSE were 0.92, 8.85%, and 10.94g, respectively.

For coconut fruit biomass measurement, the candidate independent variables included epicarp volume, total CT value of epicarp, coconut fiber volume, total CT value of coconut fiber, shell volume, total CT value of shell and other image characteristic parameters. Linear, exponential, logarithmic, polynomial and power exponential models were used for model fitting. As shown in **Figure 9E**. The Adjusted R2, MAPE, and RMSE for biomass measurement were 0.65, 14.86%, and 359.83g, respectively.

## Discussions

### The advantages and limitations of Micro-CT system

Our approach, using CT imaging of inner coconut structure with good resolution and the corresponding image analysis pipeline for coconut fruit and seed traits, provides the data required for modern coconut breeding research. In this work, we have developed a Micro-CT system to non-destructively extract coconut phenotypic traits and obtain the 3D model of the coconut fruits and seeds. There are two main series of phenotyping systems, the low-cost fast-detection systems that measure simple surface traits, and the high-cost labor-intensive systems that can obtain inner microstructures. Compared with other phenotyping methods, our approach provides a trade-off between the data richness and the acquisition difficulties. Morphology traits, such as the fruit volume and the fruit perimeter that are difficult for manual measurement can be obtained the same with the segmented 3D models. The inner structure of the fruits and seeds can be revealed without destructing the coconut shells, providing more data for coconut breeding and genomic study at a relatively low cost.

However, the limited active area of the panel detector and the relatively large size of a coconut result in an insufficient imaging space. As is shown in **Figure 5D**, the size of sample D exceeds the detector’s maximum imaging range. Thus the outermost edge of the sample cannot be observed in some of the projection images, resulting in an artifact at the corresponding position in the reconstructed slice images and affecting subsequent image processing.

### Micro-CT system reveal the structural changes in coconut seeds during germination

The Micro-CT system can obtain the inner structure of the fruits and seeds without destructing the coconut shells. It provides a stable method to examine the development status of the organs inside the coconut shell during germination. As is shown in **Figure 6**, the coconut seed sample A and sample C had a higher albumen content and larger haustorium. The albumen content in sample D is less with a smaller haustorium. On the contrary, the seedling and haustoria in sample B had only partially developed. The actual germination situation is consistent with the situation speculated from reconstructed slice images. Samples A and C successfully developed to the seedling stage; the development of sample D was slow while sample B failed to germinate. The reconstructed slice images prove that the Micro-CT system can evaluate seeds’ internal development without affecting seedlings’ development and has the potential to monitor the germination process over time.

### The potential application and extension of Micro-CT system

The system and the corresponding software are developed to measure coconut’s fruit and seed traits. Nevertheless, the applications can be easily extended to other fruits. Tropical fruits such as pitaya, mangos, and passion fruits can be measured with an appropriate tube voltage and current. Other drupes with pericarp, including peach, mango, and olive, could be accommodated with a modification of the FOV.

The difference between solid and liquid albumen in the slice images is sometimes instinctive and hard to distinguish, especially when the solid albumen layer is thin and glutinous. A dual-energy CT system can increase the contrast between different materials and improve reconstruction results. Moreover, a cone-beam CT with a circular orbit does not match Tuy’s condition. A spiral CT would be helpful to acquire the actual reconstruction result instead of an approximation.

## Conclusions

In this study, we develop a Micro-CT imaging system to extract phenotypic traits of coconuts with a high spatial resolution (up to 49 μm) and high efficiency (about 84 samples per day). A DeepLabV3+ model with an Xception backbone was trained to segment the coconut fruits and seed slice images and to establish the segmented 3D model. Up to 21 agronomic traits, together with 47 digital traits, were measured using the Micro-CT system which could be beneficial for breeding for high yield and dense planting.

## Data availability statement

The raw data supporting the conclusions of this article will be made available by the authors, without undue reservation.

## Author contributions

LY and LL designed the research, performed the experiments, analyzed the data and wrote the manuscript. WY, DW, JW, QH and ZC helped to perform the experiments. QL supervised the project and helped to design the research. All authors contributed to the article and approved the submitted version.
